# Genome Sequences of Cluster K Mycobacteriophages Deby, LaterM, LilPharaoh, Paola, SgtBeansprout, and Sulley

**DOI:** 10.1128/MRA.01481-18

**Published:** 2019-01-10

**Authors:** Joseph M. Gaballa, Keeyon Dabrian, Rachel Desai, Ryan Ngo, Diane Park, Erin Sakaji, Yiwei Sun, Boon Tan, Marcia Brinck, Olivia Brobst, Rebecca Fernando, Hannah Kim, Siobhan McCarthy, Michael Murphy, Alexandra Sarkis, Parker Sevier, Amitoj Singh, Darwin Wu, Min-Ying Wu, Hayley A. Ennis, Rohan Luhar, Justin E. Miller, Stephanie B. Orchanian, Alysha N. Salbato, Sai Alam, Lauren Brenner, Zilla Kailani, Joel Laskow, Xinyu Ma, Aika Miikeda, Paola Nol-Bernardino, Alisa Sukhina, Nikolina Walas, Wenyuan Wei, Nam Phuong Do, Christina T. Fournier, Christy J. Kim, Samantha F. Mosier, Carly Pierson, Ivonne G. Romero, Mikael Sanchez, Oyinlola Sawyerr, Joyce Wang, Rina Watanabe, Samuel Wu, Annie Chen, Katelynn Kazane, Yousif Kettoola, Emma C. Goodwin, Andrew J. Lund, William Villella, Drake Williams, Amanda Freise, Jordan Moberg Parker

**Affiliations:** a Department of Microbiology, Immunology, and Molecular Genetics, University of California, Los Angeles, Los Angeles, California, USA; b Department of Molecular, Cell, and Developmental Biology, University of California, Los Angeles, California, USA; Indiana University, Bloomington

## Abstract

Mycobacteriophages Deby, LaterM, LilPharaoh, Paola, SgtBeansprout, and Sulley were isolated from soil using Mycobacterium smegmatis mc^2^155. Genomic analysis indicated that they belong to subclusters K1 and K5. Their genomic architectures are typical of cluster K mycobacteriophages, with most variability occurring on the right end of the genome sequence.

## Abstract (precis)

Mycobacteriophages Deby, LaterM, LilPharaoh, Paola, SgtBeansprout, and Sulley were isolated from soil using Mycobacterium smegmatis mc^2^155. Genomic analysis indicated that they belong to subclusters K1 and K5.

## ANNOUNCEMENT

Mycobacteriophages, viruses that selectively infect mycobacteria, such as Mycobacterium tuberculosis and Mycobacterium leprae, are of translational interest because of their potential to counter the rising threat of antibiotic resistance among bacterial pathogens (1). The vast genetic diversity among bacteriophages allows for them to be categorized into clusters and subclusters based on nucleotide similarity (2, 3). Cluster K, which contained 119 members as of October 2018 (http://phagesdb.org/clusters/K/), is one of the only known clusters of mycobacteriophages able to infect M. tuberculosis, the pathogen responsible for tuberculosis (4). Here, we report the novel genome sequences of the five cluster K1 phages Deby, LaterM, LilPharaoh, SgtBeansprout, and Sulley and of Paola, a cluster K5 phage.

The mycobacteriophages were all isolated by enrichment cultures of Mycobacterium smegmatis mc^2^155 from soil samples collected in Los Angeles, CA, by students in the SEA-PHAGES program (5). Viral DNA was extracted with the Promega Wizard DNA clean-up kit (product number A7280), and sequencing libraries were prepared using an NEB Ultra II kit with dual-indexed barcoding or a Titanium emulsion PCR (emPCR) kit. Libraries were then pooled and run on an Illumina MiSeq platform or a GS FLX system, yielding at least 30,000 single-end reads and at least 80-fold coverage for each genome (Table 1). These reads were then assembled with Newbler version 2.9 with default settings and in each case yielded a single-phage contig, which was checked for completeness, accuracy, and phage genomic termini with Consed version 29, as previously described (6). The phage genomes were linear, double-stranded DNA with 11 nucleotide 3′ sticky overhangs. Genome sequence sizes ranged from 56,167 to 61,535 bp, and GC content varied between 65.0 and 67.1%, with an average of 66.4%, which is slightly less than the average of cluster K phages (66.9%) (Table 1).

**TABLE 1:** Novel cluster K mycobacteriophages

Phage name	Cluster	GenBank genome sequence accession no.	Sequence Read Archive raw read accession no.	Sequencing coverage (×)	Genome length (bp)	GC content (%)	No. of coding genes	No. of tRNAs
Deby	K1	MG962364	SRX5036139	4,562^*a*^	60,463	66.5	95	1
LaterM	K1	MG962371	SRX5036140	4,028^*a*^	60,143	66.5	95	1
LilPharaoh	K1	MF919518	SRX5036137	1,633^*b*^	56,167	67.1	78	0
SgtBeansprout	K1	MH020245	SRX5036138	1,052^*b*^	56,439	67.1	78	0
Sulley	K1	MF919532	SRX5036141	80^*c*^	59,873	66.4	94	1
Paola	K5	MG962374	SRX5036142	1,263^*b*^	61,535	65.0	92	1

a Sequencing performed at the NC State Genomic Sciences Laboratory (Illumina).

b Sequencing performed at the Pittsburgh Bacteriophage Institute (Illumina).

c Sequencing performed at the UCLA Genotyping and Sequencing Core (454 GS FLX pyrosequencing).

Genome annotation was performed with DNA Master (http://cobamide2.bio.pitt.edu/) and PECAAN (https://pecaan.kbrinsgd.org/), which integrate both Glimmer (7) and GeneMark (8) to predict potential open reading frames. ARAGORN (9) and tRNAscan-SE (10) were used to detect the presence of tRNAs. Gene locations were curated with Phamerator, which compares phage genes and genomes, and Starterator, which identifies conserved start sites (11). Gene functions were predicted with BLASTp (12) against the PhagesDB (13) and NCBI databases (https://www.ncbi.nlm.nih.gov/protein), as well as HHPred (14) and TMHMM (15).

Annotation revealed that the genome sequences of phages Deby, LaterM, Sulley, and Paola contained 92 to 95 coding genes and one tRNA^trp^, and LilPharaoh and SgtBeansprout had 78 coding genes and no tRNAs. In line with other cluster K mycobacteriophages, the left end of each genome was highly conserved among the phages, whereas the right end of each genome was much more variable (4) and contained the majority of genes without any known functions. The majority of the structural and the assembly genes, such as the tape measure protein and major capsid proteins, dominated the first 25 kb of the genome sequences. All phages contained lysis cassettes with lysin A, lysin B, and holin genes, and a conserved integrase was found downstream of the lysis cassettes in all phages, which suggests that they are all temperate phages potentially capable of undergoing the lysogenic life cycle.

### Data availability.

GenBank accession numbers for the six mycobacteriophages discussed in this paper are provided in Table 1.
